# Supplementary material for: Maternal prescribed opioid analgesic use during pregnancy and associations with adverse birth outcomes: A population-based study
Source: PLoS Med. 2019 Dec 2;16(12):e1002980. doi: 10.1371/journal.pmed.1002980 (PMC6886755; doi:10.1371/journal.pmed.1002980)
Supplement: S2 Appendix — (DOCX) [file pmed.1002980.s002.docx]

**S2 Appendix: Planned analyses**

*Planned analyses*

While we did not register a formal pre-specified analyses plan prior to conducting this study, the analyses presented in the paper were pre-planned, with the exception of several exploratory sensitivity analyses that we added to respond to feedback we received at conferences and during the review process. Prior to conducting this study, we described our planned approach in several grant submissions, including grants that have been funded by the National Institute on Drug Abuse and the Swedish Research Counsel. The current paper is the first paper among many that we described in the grants. For the current paper, we also followed a similar analytic strategy as the one we used in our paper on antidepressant use during pregnancy that was published in *JAMA* in 2017 [1]. Below, we have summarized our plan and where we deviated from our plans outlined in the grant proposals.

**Hypotheses**: In the grant proposals, we hypothesized:

(a) Maternal prescribed opioid analgesic (POA) use in pregnancy would be more common among women with psychiatric comorbidities, socioeconomic risk, and co-occurring pregnancy exposures and that

(b) POA use in pregnancy would be associated with increased risk of adverse offspring outcome also after adjustment for these and unmeasured potential sources of confounding, consistent with true adverse effects.

**Sample:** In the grants, we proposed analyzing a dataset created by linking several Swedish population registers, which we analyzed in our 2017 JAMA paper [1]. The grants also proposed analyzing a dataset with additional years, but this dataset is not available yet.

**Exposures:** In the grants, we outlined how we would use several indicators of POA exposure. We described how we would focus on any POA use during pregnancy, as well as the timing of exposure during pregnancy. We also explained that we would eventually utilize more fine-grained exposures, and plan to do so in future studies.

**Background characteristics:** In the grants, we described how we could select and adjust for measured traits that may confound associations between POA exposure and offspring outcomes. These traits included several pregnancy, maternal, paternal, familial, and neighborhood characteristics. We selected the same covariates as in our JAMA 2017 paper [1], plus some additional traits that would help us better index plausible confounding factors. The additional traits, which we specified in the grant proposals, were smoking during pregnancy, exposure to other psychiatric medication during pregnancy, parental substance use disorder diagnoses, parental cohabitation status at birth, neighborhood income, and neighborhood deprivation.

**Outcomes:** In the grants, we proposed studying associations with preterm birth, small for gestational age, and several other outcomes. We did not include the other outcomes in this paper because we are waiting for a larger dataset that includes offspring born through 2017.

**Main analyses to evaluate associations:** In the grants, we outlined five approaches for estimating the risk associated with prenatal POA exposure for each offspring outcome. We had previously used four out of the five of these approaches to study associations with antidepressant use during pregnancy [1].

First, we proposed ***adjusting for measured background factors*** that we found to be associated with POA use during pregnancy. Second, we described how we could use ***sibling comparisons*** to account for all unmeasured genetic and environmental factors that make siblings similar, including all maternal characteristics that remain stable across pregnancies. Third, we explained how we could use an ***active comparator*** medication, such as acetaminophen, to account for all unmeasured confounding factors that are common to the use of both types of medication, including shared indications for use. Fourth, we proposed a ***timing of exposure comparison*** in which we would compare offspring of women who filled POA prescriptions during pregnancy to offspring of women who filled POA prescriptions before pregnancy only to account for confounding by all factors shared by women with POA prescriptions around the time of pregnancy, such as having a condition for which treatment is indicated. Fifth, we proposed using ***paternal POA use in pregnancy as a negative control*** to further test the role of familial confounding. We ultimately decided not to include the paternal comparison because we now believe that several of the assumptions required in the design are not met when studying maternal POA use [e.g., 2].

**Sensitivity analyses**: We have written extensively about the assumptions of the designs we planned to use in this project [e.g., 3] and have developed several approaches to explore key limitations [e.g., 1]. Thus, the majority of the sensitivity analyses were pre-planned. However, in response to a concerns raised when presenting preliminary analyses at the Behavior Genetics Conference in June 2018, we added a sensitivity analyses evaluating the influence of exposure to medications included in combination POA medications (S9 Appendix); and, in response to reviewers’ concerns about our choice to conduct a complete-case analysis, we added a series of sensitivity analyses evaluating the role of missing data (S13 Appendix). In our grants, we also proposed to evaluate the influence of carry-over effects in sibling comparison models using case-crossover analyses; however, we decided against this approach due to confounding by maternal age and calendar time and instead tested for carry-over effects by using first-born cousin comparisons. Additionally, in the grants, we proposed conducting further sensitivity analyses to test for misclassification, which we plan to do in future studies.

[1] Sujan, A. C., Rickert, M. E., Oberg, et al. (2017). Associations of Maternal Antidepressant Use During the First Trimester of Pregnancy With Preterm Birth, Small for Gestational Age, Autism Spectrum Disorder, and Attention-Deficit/Hyperactivity Disorder in Offspring. *JAMA, 317*(15), 1553-1562.

[2] Sanderson, E., MacDonald-Wallis, C., Smith, G.D. (2018). Negative control exposure studies in the presence of measurement error: Implications for attempted effect estimate calibration. *International Journal of Epidemiology, 47*(2), 587-596.

[3] D'Onofrio BM, Class QA, Rickert ME, et al. (2016) Translational Epidemiologic Approaches to Understanding the Consequences of Early-Life Exposures. *Behavior Genetics, 46*(3), 315-328.
