# Supplementary material for: Maternal prescribed opioid analgesic use during pregnancy and associations with adverse birth outcomes: A population-based study
Source: PLoS Med. 2019 Dec 2;16(12):e1002980. doi: 10.1371/journal.pmed.1002980 (PMC6886755; doi:10.1371/journal.pmed.1002980)
Supplement: S3 Appendix — (DOCX) [file pmed.1002980.s003.docx]

**S3 Appendix: Additional information on medication exposures**

*Prescribed opioid analgesic medications*

In order to provide information on the specific POAs included in the study, Table A provides the proportions of specific types of POA medications among all POA prescriptions received by mothers during the pregnancy period. For example, of the 51,596 POA filled prescriptions filled by pregnant women between July 1^st^ 2007 and December 31^st^ 2013, 30,968 (60.05%) were codeine combinations.

In order to illustrate the types of clinics that prescribe POAs in Sweden, Table B provides the proportion of POA prescriptions prescribed by specific types of clinics among all POA prescriptions received by mothers during the pregnancy period in the year 2013. For example, of the 8,048 prescriptions filled by pregnant women in 2013, 2,698 (33.52%) originated from obstetrics and gynecology clinics, and 2,489 (30.93%) originated from primary care clinics.

In order to provide clarity on how we created the different POA exposures, Fig A illustrates the main windows for filled prescriptions we used to define use (a) before pregnancy, (b) in the washout period, (c) anytime during pregnancy, and (c) in the first trimester of pregnancy, (d) in the second trimester of pregnancy, and (e) in the third trimester of pregnancy.

*Other medications*

We included exposure to other psychoactive medication during pregnancy as a covariate in some analyses. Table C includes anatomical therapeutic chemical codes for the classes of medications we included.

Table A. Type of prescribed opioid analgesic medications received by mothers during the pregnancy period

| **Type of prescribed opioid analgesic** | **Anatomical Therapeutic Chemical Codes** | **Maternal filled prescriptions** |
| --- | --- | --- |
|  |  | **N (%)** |
| Morphine | N02AA01 | 592 (1.15) |
| Hydromorphone | N02AA03 | 3 (0.01) |
| Oxycodone | N02AA05 | 900 (1.75) |
| Oxycodone/naloxone | N02AA55 | 16 (0.03) |
| Codeine/acetaminophen | N02AA59 | 30968 (60.05) |
| Ketobemidone | N02AB01 | 212 (0.41) |
| Fentanyl | N02AB03 | 170 (0.33) |
| Dextropropoxyphene | N02AC04 | 10785 (20.91) |
| Buprenorphine | N02AE01 | 210 (0.41) |
| Morphine/antispasmodics | N02AG01 | 2948 (5.71) |
| Ketobemidone/antispasmodics | N02AG02 | 121 (0.23) |
| Tramadol | N02AX02 | 4531 (8.78) |
| Tapentadol | N02AX06 | 4 (0.01) |
| Acetaminophen/codeine | NO2BE51 | 24 (0.05) |
| Buprenorphine | N07BC01 | 37 (0.07) |
| Methadone | N07BC02 | 75 (0.15) |
| Buprenorphine, combinations | N07BC51 | 0 (0.00) |
| Total |  | 51596 (100.00) |

Table B. Types of clinics prescribing opioid analgesic medications received by mothers during the pregnancy period in 2013

|  | **Maternal filled prescriptions** |
| --- | --- |
| **Clinic type** | **N (%)** |
| Obstetrics and gynecology | 2699 (33.51) |
| Primary care | 2493 (30.95) |
| Maternity care | 1134 (14.08) |
| Surgery | 345 (4.28) |
| Non-surgical orthopedics | 230 (2.86) |
| Emergency | 201(2.50) |
| Internal medicine | 171 (2.12) |
| Anesthesiology | 109 (1.35) |
| Non-surgical dental | 109 (1.35) |
| Neurology | 70 (0.87) |
| Gastrointestinal | 53 (0.66) |
| Psychiatry | 50 (0.62) |
| Other | 341 (4.23) |
| Missing | 50 (0.62) |
| Total | 8055 (100.00) |

Table C. Other psychoactive medication classes

|  |  |
| --- | --- |
| **Medication Classes** | **Anatomical Therapeutic Chemical Codes** |
| Antidepressants | N06A |
| Benzodiazepines | N05BA, N05CD, N05CF, N03AE01 |
| Non-benzodiazepine anxiolytics | N05BB, N05BC, N05BD, N05BE, N05BX |
| Non-benzodiazepine hypnotics and sedatives | N05CA, N05CB, N05CC, N05CE, N05CH, N05CM, N05CX |
| Mood stabilizers and anticonvulsants | N03A, N05AN01 |
| Antipsychotics | N05A (excluding N05AN01) |
| Attention-deficit/hyperactivity disorder medications | N06BA01, N06BA02, N06BA04, N06BA09 |
| Substance use disorder medications | N07BA (excluding N07BA02) N07BB |

|  | | Before pregnancy | | | | | Washout | | | | Pregnancy | | | | | | | | |  | | |
| --- | --- | --- | --- | --- | --- | --- | --- | --- | --- | --- | --- | --- | --- | --- | --- | --- | --- | --- | --- | --- | --- | --- |
|  | |  |  |  |  |  |  |  |  |  | Trimester 1 | | | Trimester 2 | | | Trimester 3 | | |  | | |
|  | |  |  |  |  |  |  | | | Gestational age | | | | | | | | | |  | | |
|  | |  | | | | |  | | |  |  | | |  | | |  | | |  | | |
|  | | | |  | | | | | LMP | |  | | LMP  + 90 days | | | LMP  + 180 days | | | |  | | |
| Conception  – 360 days | | | | Conception  – 90 days | | | | | Conception | | | |  |  | | | Birth | | | | | |
| Fig A. Exposure windows. Last menstrual period (LMP) defined as birth date minus gestation age. Conception defined as LMP plus 14 days. Trimesters defined according to LMP due the clinical practice of dating pregnancy from LMP with trimester 1 defined as conception to 89 days after LMP, trimester 2 defined as 90 to 179 days after LMP, and trimester 3 defined as 180 days after LMP to birth. | | | | | | | | | | | | | | | | | | | | | | |
|  | | |  | |  |  | |  | | | |  | | |  | |  | |  | | |  |
|  | | | | | | | | | | | | | | | | |  | | |  |  |  |
