# Supplementary material for: Maternal prescribed opioid analgesic use during pregnancy and associations with adverse birth outcomes: A population-based study
Source: PLoS Med. 2019 Dec 2;16(12):e1002980. doi: 10.1371/journal.pmed.1002980 (PMC6886755; doi:10.1371/journal.pmed.1002980)
Supplement: S4 Appendix — (DOCX) [file pmed.1002980.s004.docx]

**S4 Appendix: Preliminary analyses exploring sensitive periods of exposure**

In order to evaluate whether there are sensitive periods of exposure during pregnancy, we assessed adjusted associations with maternal POA filled prescriptions occurring in only the first-trimester (conception to 89 days after last menstrual period) and only the second- or third-trimester (90 days after last menstrual to birth). We conducted Wald χ² tests to evaluate if these associations were statistically significantly different. Because these analyses intended to evaluate exposure during specific periods, we included incident users (i.e., no filled POA prescriptions in the period 360 days before pregnancy to conception) and excluded infants exposed to more than one POA during pregnancy.

In this sample of 583,686 infants born to incident users, 2,960 (0.51%) were born to women who filled a prescription only in the first trimester and 13,909 (2.38%) were born to women who filled a prescription only in the second or third trimester. The magnitude of the associations with first-trimester exposure only were slightly larger than the magnitude of associations with second/third-trimester exposure (Table A); however, the associations were not statistically significantly different for both PTB (*p*=0.25) and SGA (*p*=0.54). Therefore, these results suggest that there may not be sensitive periods of exposure during pregnancy.

Table A. Adjusted associations with exposure in the first trimester only and in the second/third trimester only in a subsample of incident users

|  |  |  | **1^st^ trimester only** | **2^nd^/3^rd^ trimester only** |
| --- | --- | --- | --- | --- |
|  |  |  | **OR (95% CI)** | **OR (95% CI)** |
| Preterm birth |  |  | 1.32 (1.13, 1.53) | 1.19 (1.10, 1.28) |
| Small for gestation age |  |  | 1.00 (0.78, 1.23) | 0.91 (0.81, 1.03) |

Note. OR=odds ratio. CI=confidence interval.
