# Supplementary material for: Maternal prescribed opioid analgesic use during pregnancy and associations with adverse birth outcomes: A population-based study
Source: PLoS Med. 2019 Dec 2;16(12):e1002980. doi: 10.1371/journal.pmed.1002980 (PMC6886755; doi:10.1371/journal.pmed.1002980)
Supplement: S5 Appendix — POA, prescribed opioid analgesic. (DOCX) [file pmed.1002980.s005.docx]

**S5 Appendix: Review of models used to evaluate associations between prescribed opioid analgesic use and birth outcomes**

In order to provide clarity on how we set up the models, Table A describes the sample used, the main predictor(s) and covariates included, and the comparison group for each main analyses model.

Table A. Main models assessing associations with prescribed opioid analgesics

| **Models assessing associations with prescribed opioid analgesics anytime during pregnancy** | | | | |
| --- | --- | --- | --- | --- |
| **Model** | **Sample** | **Main predictor** | **Other covariates** | **Comparison group** |
| Model 1: Unadjusted | Whole analytic sample | 1. Maternal POAs anytime during pregnancy | 1. Maternal POAs in washout period but not pregnancy | No maternal POAs in the washout period or pregnancy |
| Model 2:  Adjusted | Whole analytic sample | 1. Maternal POAs anytime during pregnancy | 1. Maternal POAs in washout period but not pregnancy 2. Pregnancy-related, familial, and socio-economic characteristics | No maternal POAs in the washout period or pregnancy |
| Model 3: Comparative safety | Infants with maternal POAs and/or pure acetaminophen anytime during pregnancy | 1. Maternal POAs anytime during pregnancy | 1. Maternal POAs in washout period but not pregnancy 2. Pregnancy-related, familial, and socio-economic characteristics | Maternal pure acetaminophen anytime during pregnancy and no POAs in the washout period or pregnancy |
| Model 4: Before-pregnancy-only comparison | Infants with maternal POAs sometime in the period 360 days before conception to birth. Excluded infants with POAs both before and during pregnancy. | 1. Maternal POAs anytime during pregnancy and no maternal POAs before pregnancy | 1. Maternal POAs in washout period but not pregnancy 2. Pregnancy-related, familial, and socio-economic characteristics | Maternal POAs before but not in the washout period or pregnancy |
| Model 5: Sibling comparison | Siblings that differed on POA exposure or at least one covariate. | 1. Maternal POAs anytime during pregnancy | 1. Maternal POAs in washout period but not pregnancy 2. Pregnancy-related, familial, and socio-economic characteristics | Siblings with no maternal POAs in the washout period or pregnancy |
| **Models assessing associations with prescribed opioid analgesics in a single trimester and in multiple trimesters** | | | | |
| Model 1: Unadjusted | Whole analytic sample | 1. Maternal POAs in a single trimester 2. Maternal POAs in multiple trimesters | 1. Maternal POAs in washout period but not pregnancy | No maternal POAs in the washout period or pregnancy |
| Model 2: Adjusted | Whole analytic sample | 1. Maternal POAs in a single trimester 2. Maternal POAs in multiple trimesters | 1. Maternal POAs in washout period but not pregnancy 2. Pregnancy-related, familial, and socio-economic characteristics | No maternal POAs in the washout period or pregnancy |
| Model 3a: Comparative safety model | Infants with maternal POAs and/or pure acetaminophen in a single trimester | 1. Maternal POAs in a single trimester | 1. Maternal POAs in washout period but not pregnancy 2. Pregnancy-related, familial, and socio-economic characteristics | Maternal pure acetaminophen in a single trimester and no POAs in the washout period or pregnancy |
| Model 3b: Comparative safety model | Infants with maternal POAs and/or pure acetaminophen in multiple trimesters | 1. Maternal POAs multiple trimesters | 1. Maternal POAs in washout period but not pregnancy 2. Pregnancy-related, familial, and socio-economic characteristics | Maternal pure acetaminophen in multiple trimesters and no POAs in the washout period or pregnancy |
| Model 4: Before-pregnancy-only comparison | Infants with maternal POAs sometime in the period 360 days before conception to birth. Excluded infants with POAs both before and during pregnancy. | 1. Maternal POAs in a single trimester but not before pregnancy 2. Maternal POAs in multiple trimesters but not before pregnancy | 1. Maternal POAs in washout period but not pregnancy 2. Pregnancy-related, familial, and socio-economic characteristics | Maternal POAs before pregnancy but not in the washout period or pregnancy |
| Model 5: Sibling comparison | Siblings that differed on POA exposure or at least one covariate. | 1. Maternal POAs in a single trimester 2. Maternal POAs in multiple trimesters | 1. Maternal POAs in washout period but not pregnancy 2. Pregnancy-related, familial, and socio-economic characteristics | Siblings with no maternal POAs in the washout period or pregnancy |

Note. POA=prescribed opioid analgesic
