# Supplementary material for: Maternal prescribed opioid analgesic use during pregnancy and associations with adverse birth outcomes: A population-based study
Source: PLoS Med. 2019 Dec 2;16(12):e1002980. doi: 10.1371/journal.pmed.1002980 (PMC6886755; doi:10.1371/journal.pmed.1002980)
Supplement: S6 Appendix — (DOCX) [file pmed.1002980.s006.docx]

**S6 Appendix: All parameter estimates from adjusted models estimating associations with maternal prescribed opioid filled prescriptions anytime during pregnancy**

In order to be transparent and comprehensive, we provide parameter estimates and confidence intervals for all predictors included in the adjusted models predicting PTB (Table A) and SGA (Table B) from POA use anytime during pregnancy.

Table A. Associations with preterm birth

|  | **OR (95% CI)** |
| --- | --- |
| **Maternal prescribed opioid analgesics** |  |
| During pregnancy | 1.38 (1.31, 1.45) |
| During the washout period only | 1.24 (1.12, 1.37) |
| **Pregnancy-related characteristics** |  |
| Birth order (reference: 1^st^) |  |
| 2^nd^ | 0.60 (0.58, 0.62) |
| 3^rd^ or higher | 0.68 (0.66, 0.71) |
| Year of birth 2010 to 2013 (reference: 2007 to 2009) | 0.97 (0.94, 0.99) |
| Maternal smoking during the first trimester (reference: none) |  |
| Moderate (1 to 9 cigarettes per day) | 1.21 (1.14, 1.27) |
| High (10 or more cigarettes per day) | 1.50 (1.38, 1.64) |
| Exposure to other psychoactive medications | 1.50 (1.43, 1.57) |
| **Maternal characteristics** |  |
| Opioid use disorder prior to conception | 0.89 (0.62, 1.28) |
| Non-opioid substance use disorder prior to conception | 1.00 (0.91, 1.09) |
| Schizophrenia or bipolar disorder prior to conception | 0.96 (0.81, 1.14) |
| Definite or uncertain suicide attempt prior to conception | 1.06 (0.97, 1.15) |
| Any criminal convictions prior to conception | 1.01 (0.96, 1.06) |
| Age at year of birth (reference: 20 to 29 years) |  |
| Less than 19 years | 0.98 (0.85, 1.11) |
| 30 to 39 years | 1.11 (1.08, 1.15) |
| 40 to 45 years | 1.46 (1.37, 1.55) |
| 46 years and older | 1.48 (1.08, 2.01) |
| Highest level of education at year of birth (reference: less than 9 years) |  |
| 9 years | 1.07 (0.98, 1.17) |
| 1 to 3 years upper secondary | 1.01 (0.93, 1.09) |
| Any post-secondary or postgraduate | 0.91 (0.84, 0.98) |
| Swedish nationality | 0.91 (0.87, 0.94) |
| **Paternal characteristics** |  |
| Opioid use disorder prior to conception | 1.03 (0.79, 1.35) |
| Non-opioid substance use disorder prior to conception | 0.95 (0.87, 1.04) |
| Schizophrenia or bipolar disorder prior to conception | 1.08 (0.89, 1.31) |
| Definite or uncertain suicide attempt prior to conception | 1.02 (0.92, 1.13) |
| Any criminal convictions prior to conception | 1.04 (1.01, 1.07) |
| Age at year of birth (reference: 20 to 29 years) |  |
| Less than 19 years | 1.00 (0.80, 1.25) |
| 30 to 39 years | 0.98 (0.94, 1.01) |
| 40 to 45 years | 1.01 (0.96, 1.06) |
| 46 years and older | 1.07 (1.00, 1.14) |
| Highest level of education at year of birth (reference: less than 9 years) |  |
| 9 years | 0.99 (0.91, 1.08) |
| 1 to 3 years upper secondary | 1.01 (0.93, 1.09) |
| Any post-secondary or postgraduate | 0.92 (0.84, 1.00) |
| Swedish nationality | 1.18 (1.14, 1.23) |
| **Other familial and socioeconomic characteristics** |  |
| Parents not cohabitating at birth | 0.98 (0.93, 1.04) |
| Family income at year of birth (reference: 3^rd^ quintile) |  |
| 1^st^ quintile | 1.06 (1.01, 1.12) |
| 2^nd^ quintile | 1.05 (1.01, 1.09) |
| 4^th^ quintile | 1.01 (0.98, 1.04) |
| 5^th^ quintile | 0.94 (0.90, 0.98) |
| Neighborhood deprivation at year of birth (reference: 3^rd^ quintile) |  |
| 1^st^ quintile | 1.00 (0.96, 1.05) |
| 2^nd^ quintile | 1.03 (0.98, 1.07) |
| 4^th^ quintile | 1.02 (0.98, 1.06) |
| 5^th^ quintile | 1.05 (1.01, 1.09) |

Note. OR=odds ratio. CI=confidence interval.

Table B. Associations with small for gestational age

|  | **OR (95% CI)** |
| --- | --- |
| **Maternal prescribed opioid analgesics** |  |
| During pregnancy | 1.02 (0.93, 1.10) |
| During the washout period only | 1.00 (0.86, 1.17) |
| **Pregnancy-related characteristics** |  |
| Birth order (reference: 1^st^) |  |
| 2^nd^ | 0.39 (0.37, 0.41) |
| 3^rd^ or higher | 0.36 (0.34, 0.38) |
| Year of birth 2010 to 2013 (reference: 2007 to 2009) | 0.97 (0.93, 1.00) |
| Maternal smoking during the first trimester (reference: none) |  |
| Moderate (1 to 9 cigarettes per day) | 2.02 (1.90, 2.16) |
| High (10 or more cigarettes per day) | 2.77 (2.50, 3.07) |
| Exposure to other psychoactive medications | 1.09 (1.01, 1.18) |
| **Maternal characteristics** |  |
| Opioid use disorder prior to conception | 0.91 (0.56, 1.48) |
| Non-opioid substance use disorder prior to conception | 0.97 (0.85, 1.11) |
| Schizophrenia or bipolar disorder prior to conception | 0.85 (0.66, 1.10) |
| Definite or uncertain suicide attempt prior to conception | 0.90 (0.79, 1.02) |
| Any criminal convictions prior to conception | 0.95 (0.89, 1.02) |
| Age at year of birth (reference: 20 to 29 years) |  |
| Less than 19 years | 0.64 (0.53, 0.78) |
| 30 to 39 years | 1.30 (1.24, 1.36) |
| 40 to 45 years | 1.72 (1.57, 1.88) |
| 46 years and older | 1.68 (1.08, 2.62) |
| Highest level of education at year of birth (reference: less than 9 years) |  |
| 9 years | 0.92 (0.83, 1.02) |
| 1 to 3 years upper secondary | 0.81 (0.74, 0.89) |
| Any post-secondary or postgraduate | 0.73 (0.67, 0.81) |
| Swedish nationality | 0.75 (0.71, 0.79) |
| **Paternal characteristics** |  |
| Opioid use disorder prior to conception | 1.24 (0.88, 1.73) |
| Non-opioid substance use disorder prior to conception | 0.90 (0.79, 1.02) |
| Schizophrenia or bipolar disorder prior to conception | 0.96 (0.72, 1.26) |
| Definite or uncertain suicide attempt prior to conception | 1.05 (0.91, 1.21) |
| Any criminal convictions prior to conception | 0.98 (0.93, 1.02) |
| Age at year of birth (reference: 20 to 29 years) |  |
| Less than 19 years | 1.19 (0.89, 1.58) |
| 30 to 39 years | 1.12 (1.07, 1.18) |
| 40 to 45 years | 1.24 (1.16, 1.33) |
| 46 years and older | 1.28 (1.16, 1.40) |
| Highest level of education at year of birth (reference: less than 9 years) |  |
| 9 years | 0.80 (0.73, 0.89) |
| 1 to 3 years upper secondary | 0.75 (0.69, 0.83) |
| Any post-secondary or postgraduate | 0.73 (0.66, 0.80) |
| Swedish nationality | 0.85 (0.80, 0.89) |
| **Other familial and socioeconomic characteristics** |  |
| Parents not cohabitating at birth | 1.11 (1.03, 1.19) |
| Family income at year of birth (reference: 3^rd^ quintile) |  |
| 1^st^ quintile | 1.24 (1.15, 1.32) |
| 2^nd^ quintile | 1.16 (1.06, 1.18) |
| 4^th^ quintile | 0.93 (0.88, 0.97) |
| 5^th^ quintile | 0.83 (0.78, 0.88) |
| Neighborhood deprivation at year of birth (reference: 3^rd^ quintile) |  |
| 1^st^ quintile | 1.00 (0.93, 1.07) |
| 2^nd^ quintile | 1.01 (0.95, 1.08) |
| 4^th^ quintile | 1.01 (0.96, 1.07) |
| 5^th^ quintile | 1.11 (1.05, 1.17) |

Note. OR=odds ratio. CI=confidence interval.
