# Supplementary material for: Maternal prescribed opioid analgesic use during pregnancy and associations with adverse birth outcomes: A population-based study
Source: PLoS Med. 2019 Dec 2;16(12):e1002980. doi: 10.1371/journal.pmed.1002980 (PMC6886755; doi:10.1371/journal.pmed.1002980)
Supplement: S7 Appendix — (DOCX) [file pmed.1002980.s007.docx]

**S7 Appendix: Sensitivity analyses evaluating for potential bias from exposure misclassification**

In order to evaluate for potential bias from exposure misclassification, we re-estimated the adjusted associations using a number of alternative exposure definitions.

First, we expanded the exposure window to include POA prescriptions filled in the 90 days prior to conception in case a prescription that was filled shortly before pregnancy was used during pregnancy. According to this definition, 34,758 (5.60%) infants were exposed anytime during pregnancy, 29,362 (4.73%) infants were exposed in a single trimester, and 5,396 (0.87%) were exposed in multiple trimesters.

Second, we restricted the exposure window to exclude prescriptions filled in the three days before birth to exclude prescriptions that may have been filled shortly before delivery but used only after delivery. According to this definition, 27,183 (4.38%) infants were exposed anytime during pregnancy, 22,835 (3.68%) infants were exposed in a single trimester, and 4,348 (0.70%) were exposed in multiple trimesters.

Third, in order to capture some women who may have filled POA prescription before pregnancy but used them during pregnancy, we expanded our main exposure definition to include infants with maternal-reported POA use (i.e., we defined exposure according to filled prescriptions or maternal-reported use). The Medical Birth Register contains information on maternal reports of medication use at the first antenatal visit, which typically occurs between the tenth and twelfth week of pregnancy. According to this definition, 28,467 (4.59%) infants were exposed anytime during pregnancy, 23,789 (3.83%) infants were exposed in a single trimester, and 4,678 (0.75%) were exposed in multiple trimesters.

We found commensurate results with the main analyses results using these alternative definitions (Table A), suggesting that exposure misclassification did not bias our results.

Table A. Adjusted associations with alternative exposure definitions

|  | | **Main analysis definition** | **Expanded definition** | | **Restricted definition** | **Filled prescriptions or maternal reports definition** | |
| --- | --- | --- | --- | --- | --- | --- | --- |
|  | | **OR (95% CI)** | **OR (95% CI)** | | **OR (95% CI)** | **OR (95% CI)** | |
| **Preterm birth** |  | | |  | | |  |
| Exposure anytime during pregnancy | | 1.38 (1.31, 1.45) | 1.35 (1.29, 1.41) | | 1.37 (1.30, 1.44) | 1.36 (1.29, 1.42) | |
| Exposure in a single trimester | | 1.27 (1.20, 1.34) | 1.25 (1.18, 1.31) | | 1.26 (1.19, 1.33) | 1.24 (1.17, 1.31) | |
| Exposure in multiple trimesters | | 1.97 (1.77, 2.18) | 1.89 (1.72, 2.08) | | 1.97 (1.77, 2.18) | 1.96 (1.77, 2.16) | |
| **Small for gestational age** |  | | |  | | |  |
| Exposure anytime during pregnancy | | 1.02 (0.93, 1.10) | 1.01 (0.94, 1.09) | | 1.03 (0.94, 1.12) | 1.01 (0.93, 1.10) | |
| Exposure in a single trimester | | 0.95 (0.87, 1.04) | 0.96 (0.88, 1.04) | | 0.96 (0.87, 1.06) | 0.96 (0.87, 1.05) | |
| Exposure in multiple trimesters | | 1.40 (1.17, 1.67) | 1.34 (1.14,1.58) | | 1.40 (1.17, 1.67) | 1.34 (1.12, 1.59) | |

Note. OR=odds ratio. CI=confidence interval.
