# Supplementary material for: Maternal prescribed opioid analgesic use during pregnancy and associations with adverse birth outcomes: A population-based study
Source: PLoS Med. 2019 Dec 2;16(12):e1002980. doi: 10.1371/journal.pmed.1002980 (PMC6886755; doi:10.1371/journal.pmed.1002980)
Supplement: S8 Appendix — (DOCX) [file pmed.1002980.s008.docx]

**S8 Appendix: Sensitivity analyses evaluating the type of opioid prescribed**

First, we re-estimated adjusted associations in a subsample excluding 7,287 (1.17%) infants born to mothers with during pregnancy prescriptions of dextropropoxyphene (N02AC04 and N02AC54) because this medication is no longer prescribed in Sweden.

Second, we fit models in a subsample excluding 129 (0.02%) infants born to mothers with during pregnancy prescriptions of buprenorphine (N02AE01, N07BC01, N07BC51) or methadone (N07BC02) in case these infants were exposed to prescribed opioids for the treatment of opioid use disorder rather than pain.

We found the same pattern of results as in the main analyses (Table A), suggesting that the findings are not largely driven by an opioid that is no longer prescribed in Sweden or by opioids that are prescribed for the treatment of opioid use disorder.

Table A. Adjusted associations in the whole sample, in a subsample without filled dextropropoxyphene prescriptions during pregnancy, and in a subsample without methadone or buprenorphine prescriptions

|  | **Whole sample**  **(main analyses)** | **Subsample without dextropropoxyphene** | **Subsample without filled methadone/ buprenorphine prescriptions** |
| --- | --- | --- | --- |
|  | **OR (95% CI)** | **OR (95% CI)** | **OR (95% CI)** |
| **Preterm birth** | | |  |
| Exposure anytime during pregnancy | 1.38 (1.31, 1.45) | 1.40 (1.32, 1.48) | 1.37 (1.30, 1.44) |
| Exposure in a single trimester | 1.27 (1.20, 1.34) | 1.29 (1.21, 1.37) | 1.27 (1.20, 1.34) |
| Exposure in multiple trimesters | 1.97 (1.77, 2.18) | 2.04 (1.80, 2.30) | 1.94 (1.75, 2.16) |
| **Small for gestational age** | | |  |
| Exposure anytime during pregnancy | 1.02 (0.93, 1.10) | 1.05 (0.95, 1.15) | 1.02 (0.94, 1.11) |
| Exposure in a single trimester | 0.95 (0.87, 1.04) | 0.95 (0.85, 1.06) | 0.95 (0.87, 1.05) |
| Exposure in multiple trimesters | 1.40 (1.17, 1.67) | 1.63 (1.34, 1.98) | 1.42 (1.19, 1.69) |

Note. OR=odds ratio. CI=confidence interval.
