# Supplementary material for: Maternal prescribed opioid analgesic use during pregnancy and associations with adverse birth outcomes: A population-based study
Source: PLoS Med. 2019 Dec 2;16(12):e1002980. doi: 10.1371/journal.pmed.1002980 (PMC6886755; doi:10.1371/journal.pmed.1002980)
Supplement: S9 Appendix — POA, prescribed opioid analgesic. (DOCX) [file pmed.1002980.s009.docx]

**S9 Appendix: Sensitivity analyses evaluating influence of inclusion of combination prescribed opioid analgesic medications**

In order to evaluate whether exposure to medications other than POAs that are included in POA combination medications influenced the main analyses results, we re-estimated adjusted associations in a subsample excluding 19,697 (3.17%) infants born to mothers with filled prescriptions of combination POA medications (i.e., oxycodone/naloxone [N02AA55], buprenorphine/naloxone [N07BC51], morphine/antispasmodics [N02AG01], ketobemidone/antispasmodics [N02AG02], hydromorphone/antispasmodics [N02AG04], codeine combinations [N02AA59], and dextropropoxyphene combinations [N02AC54]).

We found the same pattern of results using this subsample as in the main analyses (Table A), suggesting that the results were not driven by inclusion of combination POA medications.

Table A. Adjusted associations in the whole sample and in a subsample without during pregnancy filled prescriptions of combination prescribed opioid analgesic medications

|  | **Whole sample**  **(main analyses)** | **Subsample without combination prescribed opioid analgesic medications** |
| --- | --- | --- |
|  | **OR (95% CI)** | **OR (95% CI)** |
| **Preterm birth** | | |
| Exposure anytime during pregnancy | 1.38 (1.31, 1.45) | 1.33 (1.21, 1.45) |
| Exposure in a single trimester | 1.27 (1.20, 1.34) | 1.27 (1.15, 1.41) |
| Exposure in multiple trimesters | 1.97 (1.77, 2.18) | 1.66 (1.33, 2.08) |
| **Small for gestational age** | | |
| Exposure anytime during pregnancy | 1.02 (0.93, 1.10) | 0.98 (0.84, 1.14) |
| Exposure in a single trimester | 0.95 (0.87, 1.04) | 0.95 (0.82, 1.15) |
| Exposure in multiple trimesters | 1.40 (1.17, 1.67) | 1.16 (0.79, 1.71) |

Note. OR=odds ratio. CI=confidence interval.
