# Supplementary material for: Maternal prescribed opioid analgesic use during pregnancy and associations with adverse birth outcomes: A population-based study
Source: PLoS Med. 2019 Dec 2;16(12):e1002980. doi: 10.1371/journal.pmed.1002980 (PMC6886755; doi:10.1371/journal.pmed.1002980)
Supplement: S10 Appendix — (DOCX) [file pmed.1002980.s010.docx]

**S10 Appendix: Sensitivity analyses evaluating the role of polypharmacy**

In our sample, approximately 15% of POA-exposed infants versus 4% of POA-unexposed infants were exposed to another psychoactive medication during pregnancy (Table 2). Therefore, it is possible that maternal use of additional psychoactive medications accounted for observed associations between POA exposure and increased risk for adverse birth outcomes in infants. Although we included polypharmacy as a covariate in the main analyses, to further assess whether exposure to polypharmacy was responsible for observed associations with POA exposure, we re-estimated adjusted associations in a subsample excluding 40,050 (6.45%) infants born to women prescribed other psychoactive medications during pregnancy. The results were commensurate with main analyses results (Table A), suggesting that associations with POA exposure observed in main analyses were not driven by exposure to polypharmacy.

Table A. Adjusted associations in a subsample without polypharmacy

|  | **Adjusted associations in the whole sample**  **(main analyses)** | | **Adjusted associations in a subsample without polypharmacy** |
| --- | --- | --- | --- |
|  | **OR (95% CI)** | | **OR (95% CI)** |
| **Preterm birth** | |  |  |
| Exposure anytime during pregnancy | 1.38 (1.31, 1.45) | | 1.33 (1.25, 1.41) |
| Exposure in a single trimester | 1.27 (1.20, 1.34) | | 1.26 (1.18, 1.34) |
| Exposure in multiple trimesters | 1.97 (1.77, 2.18) | | 1.87 (1.63, 2.15) |
| **Small for gestational age** | |  |  |
| Exposure anytime during pregnancy | 1.02 (0.93, 1.10) | | 1.01 (0.92, 1.10) |
| Exposure in a single trimester | 0.95 (0.87, 1.04) | | 0.95 (0.86, 1.06) |
| Exposure in multiple trimesters | 1.40 (1.17, 1.67) | | 1.39 (1.00, 1.75) |

Note. OR=odds ratio. CI=confidence interval.
