# Supplementary material for: Maternal prescribed opioid analgesic use during pregnancy and associations with adverse birth outcomes: A population-based study
Source: PLoS Med. 2019 Dec 2;16(12):e1002980. doi: 10.1371/journal.pmed.1002980 (PMC6886755; doi:10.1371/journal.pmed.1002980)
Supplement: S11 Appendix — (DOCX) [file pmed.1002980.s011.docx]

**S11 Appendix: Sensitivity analyses evaluating assumptions of sibling comparisons results**

We performed two analyses to evaluate assumptions of sibling comparison designs.

First, to assess if the sibling comparison results would generalize to the entire population, which includes families without siblings, we estimated adjusted population-wide associations in a sample of 288,995 infants who had siblings in the dataset. We found population-wide associations in the subsample of siblings were commensurate to population-wide associations in the entire sample (Table A), suggesting that siblings comparison results would generalize to families without siblings.

Second, to evaluate if carry-over effects (i.e., exposure in a prior pregnancy affecting subsequent pregnancies) influenced the sibling comparison results, we compared the risk for the outcomes among first-born cousins. The first-born cousin comparison results were comparable to the sibling comparison results (Table B), suggesting that carry-over effects did not account for similar risk of the outcomes among differentially exposed siblings.

Table A. Adjusted population wide associations among all infants and infants with siblings in the sample

|  | **All infants**  **(main analyses)** | **Siblings** |
| --- | --- | --- |
|  | **OR (95% CI)** | **OR (95% CI)** |
| **Preterm birth** |  |  |
| Exposure anytime during pregnancy | 1.38 (1.31, 1.45) | 1.33 (1.23, 1.45) |
| Exposure in a single trimester | 1.27 (1.20, 1.34) | 1.28 (1.17, 1.39) |
| Exposure in multiple trimesters | 1.97 (1.77, 2.18) | 1.69 (1.41, 2.03) |
| **Small for gestational age** |  |  |
| Exposure anytime during pregnancy | 1.02 (0.93, 1.10) | 1.04 (0.90, 1.19) |
| Exposure in a single trimester | 0.95 (0.87, 1.04) | 0.99 (0.85, 1.15) |
| Exposure in multiple trimesters | 1.40 (1.17, 1.67) | 1.34 (0.97, 1.84) |

Note. OR=odds ratio. CI=confidence interval.

Table B. Sibling comparison and first-born cousin comparison associations

|  | **Sibling comparison (main analyses)** | **First-born cousin comparison** |
| --- | --- | --- |
|  | **OR (95% CI)** | **OR (95% CI)** |
| **Preterm birth** |  |  |
| Exposure anytime during pregnancy | 0.99 (0.85, 1.14) | 1.01 (0.72, 1.40) |
| Exposure in a single trimester | 0.99 (0.85, 1.15) | 1.01 (0.70, 1.44) |
| Exposure in multiple trimesters | 1.04 (0.70, 1.55) | 1.03 (0.44, 2.41) |
| **Small for gestational age** |  |  |
| Exposure anytime during pregnancy | 0.91 (0.70, 1.19) | 0.84 (0.54, 1.31) |
| Exposure in a single trimester | 0.90 (0.69, 1.18) | 0.79 (0.48, 1.28) |
| Exposure in multiple trimesters | 1.22 (0.60, 2.48) | 1.17 (0.38, 3.61) |

Note. OR=odds ratio. CI=confidence interval.
