# Supplementary material for: Maternal prescribed opioid analgesic use during pregnancy and associations with adverse birth outcomes: A population-based study
Source: PLoS Med. 2019 Dec 2;16(12):e1002980. doi: 10.1371/journal.pmed.1002980 (PMC6886755; doi:10.1371/journal.pmed.1002980)
Supplement: S12 Appendix — (DOCX) [file pmed.1002980.s012.docx]

**S12 Appendix: Sensitivity analyses assessing associations with continuous outcomes**

In order to evaluate whether an influence of POA exposure was not reflected in outcomes based on clinical cut-off values, we fit all main models predicting birth outcomes on continuous scales (i.e., gestation age [mean=278.79 days; standard deviation=12.19 days] and birth weight [mean=3552.51 grams; standard deviation=551.04 grams] adjusted for gestational age.

The results were consistent with the pattern of findings for dichotomous outcomes (Table A). POA exposure was associated with reduced gestational age in unadjusted models and the associations were attenuated in subsequent models. For example, infants exposed in multiple trimesters, on average were born 5 days earlier than unexposed infants; however, siblings exposed in multiple trimesters had gestational periods that were less than 1 day shorter (0.73) than their unexposed siblings. These results suggest that confounding factors explain most of the observed associations between prenatal POA exposure and reduced gestational age. However, these analyses do not rule out a small causal effect of POA exposure on reducing gestational age.

Although infants exposed to POAs in multiple trimesters were on average 24 grams lighter than infants exposed to acetaminophen in multiple trimesters, across all other models POA exposure was not statistically significantly associated with reduced birth weight. These results suggest that POA exposure is unlikely to considerably increase the risk for reduced fetal growth.

Table A. Associations with continuous outcomes

|  | | **Model 1:**  **Unadjusted** | **Model 2:**  **Adjusted** | | **Model 3:**  **Comparative safety** | **Model 4:**  **Before-pregnancy-only comparison** | | **Model 5:**  **Sibling comparison** |
| --- | --- | --- | --- | --- | --- | --- | --- | --- |
|  | | ***B* (95% CI)** | ***B*(95% CI)** | | ***B* (95% CI)** | ***B* (95% CI)** | | ***B* (95% CI)** |
| **Gestational age (days)** |  | | |  | | |  | |
| Exposure anytime during pregnancy | | -2.45 (-2.59, -2.30) | -1.95 (-2.10, -1.81) | | -1.10 (-1.38, -0.81) | -0.46 (-0.71, -0.22) | | -0.33 (-0.60, -0.06) |
| Exposure in a single trimester | | -1.93 (-2.09, -1.77) | -1.93 (-2.09, -1.77) | | 0.11 (-0.16, 0.39) | -0.27 (-0.52, -0.01) | | -0.31 (-0.58, -0.03) |
| Exposure in multiple trimesters | | -5.18 (-5.54, -4.82) | -5.18 (-5.54, -4.82) | | -2.38 (-2.81, -1.95) | -2.35 (-2.92, -1.77) | | -0.73 (-1.49, 0.03) |
| **Birth weight (grams) adjusted for gestational age** | | | | | | | | |
| Exposure anytime during pregnancy | | 30.39 (25.00, 35.78) | 21.60 (16.34, 26.85) | | 11.49 (1.79, 21.19) | 4.73 (-3.89, 13.35) | | 4.81 (-3.67, 13.29) |
| Exposure in a single trimester | | 33.66 (27.82, 39.51) | 26.22 (20.54, 31.90) | | 19.30 (9.93, 28.67) | 5.64 (-3.19, 14.46) | | 5.53 (-3.18, 14.24) |
| Exposure in multiple trimesters | | 12.85 (-0.46, 26.16) | -5.67 (-18.64, 7.31) | | -24.40 (-39.12, -9.69) | -4.09 (-24.32, 16.14) | | -4.15 (-27.98, 19.69) |

Note. *B*=beta weight. CI=confidence interval.
