# Supplementary material for: Maternal prescribed opioid analgesic use during pregnancy and associations with adverse birth outcomes: A population-based study
Source: PLoS Med. 2019 Dec 2;16(12):e1002980. doi: 10.1371/journal.pmed.1002980 (PMC6886755; doi:10.1371/journal.pmed.1002980)
Supplement: S13 Appendix — (DOCX) [file pmed.1002980.s013.docx]

**S13 Appendix: Sensitivity analyses evaluating the influence of missing data**

Our target sample included 688,932 infants. We excluded approximately 10% of this sample (68,474 infants) with missing data on covariates from the target sample to create the analytic sample. In response to review’s feedback, we conducted sensitivity analyses to evaluate the influence of missing data.

First, we estimated covariate adjusted associations between absence of data and POA exposure in the target sample. Absence of data was not associated with prescriptions anytime during pregnancy after adjusting for all measured covariates in our target sample (OR=1.03, 95% CI:0.98-1.08), indicating that covariate adjustment helped minimize potential bias from missing data.

Second, in the analytic sample, we only adjusted for covariates with complete data. These analyses showed commensurate results to the main analyses adjusted associations (Table A). Then, we again only adjusted for covariates with complete data, but this time we conducted the analyses in the target sample with no missing data. Again, we found commensurate associations (Table A). These results indicate that excluding infants with missing covariate data did not bias our results.

Table A. Adjusted associations in the target and analytic samples

|  | **Adjusted for all covariates in the analytic sample (main analyses)** | **Adjusted only for covariates with complete data^a^ in the target sample** | **Adjusted only for covariates with complete data^a^ in the analytic sample** |
| --- | --- | --- | --- |
|  | **OR (95% CI)** | **OR (95% CI)** | **OR (95% CI)** |
| **Preterm birth** |  |  |  |
| Exposure anytime during pregnancy | 1.38 (1.31, 1.45) | 1.40 (1.33, 1.46) | 1.42 (1.35, 1.49) |
| Exposure in a single trimester | 1.27 (1.20, 1.34) | 1.29 (1.22, 1.36) | 1.30 (1.22, 1.37) |
| Exposure in multiple trimesters | 1.97 (1.77, 2.18) | 1.97 (1.79, 2.18) | 2.07 (1.87, 2.30) |
| **Small for gestational age** |  |  |  |
| Exposure anytime during pregnancy | 1.02 (0.93, 1.10) | 1.06 (0.98, 1.14) | 1.06 (0.98, 1.15) |
| Exposure in a single trimester | 0.95 (0.87, 1.04) | 0.98 (0.90, 1.07) | 0.98 (0.89, 1.08) |
| Exposure in multiple trimesters | 1.40 (1.17, 1.67) | 1.48 (1.26, 1.75) | 1.50 (1.26, 1.79) |

Note. OR=odds ratio. CI=confidence interval. **^a^**Models adjusted for parity; year of birth; exposure to other psychiatric medications during pregnancy; maternal opioid use disorder, non-opioid substance use disorder, schizophrenia or bipolar disorder, definite or uncertain suicide attempt, and any criminal conviction before conception; and maternal age at childbearing.
