# Supplementary material for: Maternal prescribed opioid analgesic use during pregnancy and associations with adverse birth outcomes: A population-based study
Source: PLoS Med. 2019 Dec 2;16(12):e1002980. doi: 10.1371/journal.pmed.1002980 (PMC6886755; doi:10.1371/journal.pmed.1002980)
Supplement: S14 Appendix — (DOCX) [file pmed.1002980.s014.docx]

**S14 Appendix: Prevalence of the covariates stratified by exposure status in the target sample**

In Table 1, we reported prevalence of background characteristics among unexposed infants and infants exposed to POAs anytime during pregnancy. Table A also reports background characteristics among infants exposed to POAs in a single-trimester and infants exposed to POAs in multiple-trimesters.

Table A. Prevalence of the covariates stratified by exposure status in the target sample

|  | **Unexposed** | **Ever Exposed** | **Single-trimester exposure** | **Multiple-trimester exposure** |
| --- | --- | --- | --- | --- |
|  | **(n=658,580 [95.59%])** | **(n=30,352 [4.41%])** | **(n=25,544 [3.71%])** | **(n=4,808 [0.70%])** |
|  | **N (%)** | **N (%)** | **N (%)** | **N (%)** |
| **Pregnancy-related characteristics** | | | | |
| Birth order |  |  |  |  |
| 1^st^ (reference) | 296455 (45.01) | 11883 (39.15) | 10275 (40.22) | 1608 (33.44) |
| 2^nd^ | 241854 (36.72) | 10884 (35.86) | 9230 (36.13) | 1654 (34.40) |
| 3^rd^ or higher | 120271 (18.26) | 7585 (24.99) | 6039 (23.64) | 1546 (32.15) |
| Year of birth |  |  |  |  |
| 2007 to 2009 (reference) | 246792 (37.47) | 11016 (36.29) | 9195 (36.00) | 1821 (37.87) |
| 2010 to 2013 | 411788 (62.53) | 19336 (63.71) | 16349 (64.00) | 2987 (62.13) |
| Maternal smoking during the first trimester |  |  |  |  |
| None (reference) | 596358 (90.55) | 25881 (85.27) | 22143 (86.69) | 3738 (77.75) |
| Moderate (1 to 9 cigarettes per day) | 30513 (4.63) | 2455 (8.09) | 1852 (7.25) | 603 (12.54) |
| High (10 or more cigarettes per day) | 8409 (1.28) | 918 (3.02) | 648 (2.54) | 270 (5.62) |
| Missing | 23300 (3.54) | 1098 (3.62) | 901 (3.53) | 197 (4.10) |
| Exposure to other psychoactive medications | 28432 (4.32) | 4695 (15.47) | 3938 (15.42) | 1814 (37.73) |
| **Maternal characteristics** | | | | |
| Opioid use disorder before conception | 468 (0.07) | 123 (0.41) | 54 (0.21) | 69 (1.44) |
| Non-opioid substance use disorder before conception | 10298 (1.56) | 960 (3.16) | 683 (2.67) | 277 (5.76) |
| Schizophrenia or bipolar disorder before conception | 2576 (0.39) | 285 (0.94) | 217 (0.85) | 68 (1.41) |
| Definite or uncertain suicide attempt before conception | 11181 (1.70) | 1247 (4.11) | 926 (3.63) | 321 (6.68) |
| Any criminal convictions before conception | 43190 (6.56) | 3352 (11.04) | 2520 (9.87) | 832 (17.30) |
| Age at year of birth |  |  |  |  |
| Less than 19 years | 6797 (1.03) | 188 (0.62) | 168 (0.66) | 20 (0.42) |
| 20 to 29 years (reference) | 256894 (39.01) | 11526 (37.97) | 9901 (38.76) | 1625 (33.80) |
| 30 to 39 years | 362436 (55.03) | 16858 (55.54) | 14054 (55.02) | 2804 (58.32) |
| 40 to 45 years | 31600 (4.80) | 1736 (5.72) | 1384 (5.42) | 352 (7.32) |
| 46 years and older | 853 (0.13) | 44 (0.14) | 37 (0.14) | 7 (0.15) |
| Highest level of education at year of birth |  |  |  |  |
| Less than 9 years (reference) | 19895 (3.02) | 865 (2.85) | 755 (2.96) | 110 (2.29) |
| 9 years | 50739 (7.70) | 3729 (12.29) | 2944 (11.53) | 785 (16.33) |
| 1 to 3 years upper secondary | 239629 (36.39) | 13022 (42.90) | 10739 (42.04) | 2283 (47.48) |
| Any post-secondary or postgraduate | 332690 (50.52) | 12301 (40.53) | 10732 (42.01) | 1569 (32.63) |
| Missing | 15629 (2.37) | 435 (1.43) | 374 (1.46) | 61 (1.27) |
| Country of origin |  |  |  |  |
| Sweden | 502636 (76.32) | 24079 (79.33) | 19997 (78.28) | 4082 (84.90) |
| Missing | 98 (0.01) | 1 (0.00) | 1 (0.00) | 0 (0.00) |
| **Paternal characteristics** | | | | |
| Opioid use disorder before conception | 984 (0.15) | 102 (0.34) | 72 (0.27) | 32 (0.67) |
| Non-opioid substance use disorder before conception | 11554 (1.75) | 832 (2.74) | 635 (2.49) | 197 (4.10) |
| Schizophrenia or bipolar disorder before conception | 1968 (0.30) | 144 (0.47) | 110 (0.43) | 34 (0.71) |
| Definite or uncertain suicide attempt before conception | 7980 (1.21) | 474 (1.56) | 379 (1.48) | 95 (1.98) |
| Any criminal convictions before conception | 120387 (18.28) | 7117 (23.45) | 5698 (22.31) | 1419 (29.51) |
| Age at year of birth |  |  |  |  |
| Less than 19 years | 2023 (0.31) | 67 (0.22) | 61 (0.24) | 6 (0.12) |
| 20 to 29 years (reference) | 157717 (23.95) | 7352 (24.22) | 6280 (24.59) | 1072 (22.30) |
| 30 to 39 years | 377775 (57.36) | 17021 (56.08) | 14350 (56.18) | 2671 (55.55) |
| 40 to 45 years | 79403 (12.06) | 3939 (12.98) | 3221 (12.61) | 718 (14.93) |
| 46 years and older | 27724 (4.21) | 1346 (4.43) | 1121 (4.39) | 225 (4.68) |
| Missing | 13938 (2.12) | 627 (2.07) | 511 (2.00) | 116 (2.41) |
| Highest level of education at year of birth |  |  |  |  |
| Less than 9 years (reference) | 17865 (2.71) | 788 (2.60) | 681 (2.67) | 107 (2.23) |
| 9 years | 60313 (9.16) | 3432 (11.31) | 2778 (10.88) | 654 (13.60) |
| 1 to 3 years upper secondary | 293777 (44.61) | 15014 (49.47) | 12514 (48.99) | 2500 (52.00) |
| Any post-secondary or postgraduate | 259281 (39.37) | 9985 (32.90) | 8620 (33.75) | 1365 (28.39) |
| Missing | 27344 (4.15) | 1133 (3.73) | 951 (3.72) | 182 (3.79) |
| Country of origin |  |  |  |  |
| Sweden | 492997 (74.86) | 23367 (76.99) | 19486 (76.28) | 3881 (80.72) |
| Missing | 14042 (2.13) | 629 (2.07) | 512 (2.00) | 117 (2.43) |
| **Other familial and socioeconomic characteristics** | | | | |
| Parental cohabitation status at birth |  |  |  |  |
| Parents not cohabitating at birth | 39845 (6.05) | 2302 (7.58) | 1839 (7.20) | 463 (9.63) |
| Missing | 24936 (3.79) | 1194 (3.93) | 998 (3.91) | 196 (4.08) |
| Family income at year of birth |  |  |  |  |
| 1^st^ quintile (lowest income) | 55036 (8.36) | 2183 (7.19) | 1798 (7.04) | 385 (8.01) |
| 2^nd^ quintile | 85747 (13.02) | 4319 (14.23) | 3575 (14.00) | 744 (15.47) |
| 3^rd^ quintile (reference) | 190101 (28.87) | 9292 (30.61) | 7757 (30.37) | 1535 (31.93) |
| 4^th^ quintile | 222018 (33.71) | 10098 (33.27) | 8555 (33.49) | 1543 (32.09) |
| 5^th^ quintile (highest income) | 105678 (16.05) | 4460 (14.69) | 3859 (15.11) | 601 (12.50) |
| Missing | 2005 (0.30) | 39 (0.13) | 34 (0.13) | 5 (0.10) |
| Neighborhood deprivation at year of birth |  |  |  |  |
| 1^st^ quintile (least neighborhood deprivation) | 96203 (14.61) | 4179 (13.77) | 3534 (13.83) | 645 (13.42) |
| 2^nd^ quintile | 113124 (17.18) | 5037 (16.60) | 4248 (16.63) | 789 (16.41) |
| 3^rd^ quintile (reference) | 118578 (18.01) | 5379 (17.72) | 4540 (17.77) | 839 (17.45) |
| 4^th^ quintile | 140301 (21.30) | 6532 (21.52) | 5444 (21.31) | 1088 (22.63) |
| 5^th^ quintile (most neighborhood deprivation) | 190374 (28.91) | 9225 (30.39) | 7778 (30.45) | 1447 (30.10) |
| Missing | 1189 (0.18) | 43 (0.14) | 34 (0.13) | 9 (0.19) |
