# Supplementary material for: Maternal prescribed opioid analgesic use during pregnancy and associations with adverse birth outcomes: A population-based study
Source: PLoS Med. 2019 Dec 2;16(12):e1002980. doi: 10.1371/journal.pmed.1002980 (PMC6886755; doi:10.1371/journal.pmed.1002980)
Supplement: S15 Appendix — PTB, preterm birth; SGA, small for gestational age. (DOCX) [file pmed.1002980.s015.docx]

**S15 Appendix: Prevalence of PTB and SGA among all exposure and comparison groups**

In order to help interpret main analysis odds ratios, Table A presents prevalence of PTB and SGA among exposure and comparison groups.

Table A. Prevalence of preterm birth and small for gestation age infants among all exposure and comparison groups

|  | **Exposed to prescribed opioid analgesics during pregnancy**  **(n=27,559)** | **Unexposed to prescribed opioid analgesics during pregnancy**  **(n=592,899)** | **Exposed to pure acetaminophen during pregnancy**  **(n=13,116)** | **^a^Exposed to prescribed opioid analgesics before pregnancy only**  **(n=18,883)** | **Sibling exposed to prescribed opioid analgesics during pregnancy**  **(n=9,386)** | **Sibling unexposed to prescribed opioid analgesics during pregnancy**  **(n=10,103)** |
| --- | --- | --- | --- | --- | --- | --- |
|  | **N (%)** | **N (%)** | **N (%)** | **N (%)** | **N (%)** | **N (%)** |
| Preterm birth | 1771 (6.43) | 26323 (4.44) | 703 (5.36) | 1061 (5.62) | 481 (5.12) | 544 (5.38) |
| Small for gestational age | 694 (2.19) | 12519 (2.11) | 317 (2.42) | 415 (2.20) | 158 (1.68) | 202 (2.00) |
