# Supplementary material for: Maternal prescribed opioid analgesic use during pregnancy and associations with adverse birth outcomes: A population-based study
Source: PLoS Med. 2019 Dec 2;16(12):e1002980. doi: 10.1371/journal.pmed.1002980 (PMC6886755; doi:10.1371/journal.pmed.1002980)
Supplement: Attachment — Submitted filename: POA_BO_PLOSMed_response_to_reviews_9.23.19.docx [file pmed.1002980.s016.docx]

Clare Stone, Ph.D.

Acting Editor in Chief

*PLOS Medicine*

September 23, 2019

Dear Dr. Stone,

On behalf of my co-authors, I am pleased to submit a revised version of our manuscript now entitled “Maternal prescribed opioid analgesic use during pregnancy and associations with adverse birth outcomes: A population-based study.” We are thrilled that *PLOS Medicine* will consider a revised version of our paper and are grateful for the insightful feedback you and the four reviewers provided. We have incorporated the suggestions and believe the resulting manuscript is substantially improved. We also have provided point-by-point responses to each comment below. Of note, we added a sensitivity analyses to address reviewer concerns about missing data. Given that our supplemental material was already quite long, we removed our sensitivity analyses replicating results using single versus multiple POA prescriptions during pregnancy. As requested, we have also included a version of the manuscript with track changes highlighted, as well as a “clean” version.

Sincerely,

Ayesha Sujan, M.A.

Graduate Student, Clinical Science

Department of Psychological and Brain Sciences

Indiana University

1101 E. 10th St.

Bloomington, IN 47405

Phone: 812-856-2588

asujan@indiana.edu

**Requests from the editors:**

Title – association with, instead of risk, please.

*Response: We have made this change (line 1).*

Abstract – paragraph one you say “if observed associations” but don’t tell us what the associations are’; similarly on first mention of the 2 birth outcomes please do say what they are; The abstract is very long, I would suggest cutting the paragraph starting with ‘across all models..’; please provide a sentence on the limitations of the study as the final sentence of the

Methods and Findings section of the abstract; please provide some summary demographic information on participants.

*Response: We made several changes to the abstract to address these concerns. First, we edited the paragraph to make it clear that we are referring to associations between POA exposure during pregnancy and birth outcomes (line 24-25). Second, we added the specific outcomes we studied to the end of the background section (line 29-30). Third, we simplified and shortened the abstract by removing some details about single versus multiple-trimester exposure and associations between any POA use during pregnancy and SGA. Fourth, we added a sentence on study limitations (line 78-80). Fifth, we now include information on the percentage of female and first-born offspring (line 35).*

Data – please provide a link or email address for statistics Sweden.

*Response: We have included a citation in the method section (174) that points to the reference with a link (line 1047).*

At this stage, we ask that you include a short, non-technical Author Summary of your research to make findings accessible to a wide audience that includes both scientists and non-scientists.

The Author Summary should immediately follow the Abstract in your revised manuscript. This text is subject to editorial change and should be distinct from the scientific abstract. Please see our author guidelines for more information: <https://journals.plos.org/plosmedicine/s/revising-your-manuscript#loc-author-summary>

*Response: We have added an author summary (line 106-126).*

Throughout inc tables, please provide p values where any data is quantified (already with 95%Cis)

*Response: We have provided p-values for all main analyses below, for reference. We also note the longstanding concerns about misguided focus on statistical significance in research [1,2] and that adding p-values to our publication would conflict with our efforts to heed recent recommendations from the American Statistical Association [3] (accompanied by a forceful commentary in Nature, supported by over 800 signatories [4]).*

*1. Significance questing. Rothman KJ. Editorial in Annals of Internal Medicine 1986*

*2. Sifting the evidence – what’s wrong with significance tests? Sterne J, Davey-Smith G. Comment in BMJ 2001*

*3. Moving to a World Beyond “p < 0.05”. Wasserstein RL, Schirm A, Lazar NA. Editorial in the American Statistician 2019*

*4. Retire statistical significance. Amrhein V, Greenland S, McShane S. Comment in Nature 2019*

P-values for demographics table

|  | ***P*-value** |
| --- | --- |
| **Pregnancy-related characteristics** |  |
| Birth order |  |
| 1^st^ | Reference |
| 2^nd^ | <.0001 |
| 3^rd^ or higher | <.0001 |
| Year of birth |  |
| 2007 to 2009 | Reference |
| 2010 to 2013 | <.0001 |
| Maternal smoking during the first-trimester |  |
| None | Reference |
| Moderate (1 to 9 cigarettes per day) | <.0001 |
| High (10 or more cigarettes per day) | <.0001 |
| Missing | .009 |
| Exposure to other psychoactive medications | <.0001 |
| **Maternal characteristics** |  |
| Opioid use disorder before conception | <.0001 |
| Non-opioid substance use disorder before conception | <.0001 |
| Schizophrenia or bipolar disorder before conception | <.0001 |
| Definite or uncertain suicide attempt before conception | <.0001 |
| Any criminal convictions before conception | <.0001 |
| Age at year of birth |  |
| Less than 19 years | <.0001 |
| 20 to 29 years | Reference |
| 30 to 39 years | 0.004 |
| 40 to 45 years | <.0001 |
| 46 years and older | 0.37 |
| Highest level of education at year of birth |  |
| Less than 9 years | Reference |
| 9 years | <.0001 |
| 1 to 3 years upper secondary | <.0001 |
| Any post-secondary or postgraduate | <.0001 |
| Missing | <.0001 |
| Country of origin |  |
| Sweden | <.0001 |
| Missing | 0.174 |
| **Paternal characteristics** |  |
| Opioid use disorder before conception | <.0001 |
| Non-opioid substance use disorder before conception | <.0001 |
| Schizophrenia or bipolar disorder before conception | <.0001 |
| Definite or uncertain suicide attempt before conception | <.0001 |
| Any criminal convictions before conception | <.0001 |
| Age at year of birth |  |
| Less than 19 years | 0.006 |
| 20 to 29 years | Reference |
| 30 to 39 years | 0.017 |
| 40 to 45 years | 0.002 |
| 46 years and older | 0.180 |
| Missing | 0.403 |
| Highest level of education at year of birth |  |
| Less than 9 years | Reference |
| 9 years | <.0001 |
| 1 to 3 years upper secondary | <.0001 |
| Any post-secondary or postgraduate | 0.0003 |
| Missing | 0.187 |
| Country of origin |  |
| Sweden | <.0001 |
| Missing | 0.124 |
| **Other familial and socioeconomic characteristics** |  |
| Parental cohabitation status at birth |  |
| Parents not cohabitating at birth | <.0001 |
| Missing | 0.060 |
| Family income at year of birth |  |
| 1^st^ quintile (lowest income) | <.0001 |
| 2^nd^ quintile | 0.112 |
| 3^rd^ quintile | Reference |
| 4^th^ quintile | <.0001 |
| 5^th^ quintile (highest income) | <.0001 |
| Missing | <.0001 |
| Neighborhood deprivation at year of birth |  |
| 1^st^ quintile (least neighborhood deprivation) | 0.051 |
| 2^nd^ quintile | 0.353 |
| 3^rd^ quintile | Reference |
| 4^th^ quintile | 0.236 |
| 5^th^ quintile (most neighborhood deprivation) | 0.0002 |
| Missing | 0.236 |

*P*-value for main analyses associations between prescribed opioid analgesic exposure and adverse birth outcomes

|  | **Model 1:**  **Unadjusted** | **Model 2:**  **Adjusted** | **Model 3:**  **Comparative safety** | **Model 4:**  **Before-pregnancy-only comparison** | **Model 5:**  **Sibling comparison** |
| --- | --- | --- | --- | --- | --- |
|  | ***P*-value** | ***P*-value** | ***P*-value** | ***P*-value** | ***P*-value** |
| **Preterm birth** |  |  |  |  |  |
| Exposure anytime during pregnancy | <.0001 | <.0001 | 0.001 | 0.266 | 0.919 |
| Exposure in a single trimester | <.0001 | <.0001 | 0.027 | 0.987 | 0.874 |
| Exposure in multiple trimesters | <.0001 | <.0001 | <.0001 | <.0001 | 0.830 |
| **Small for gestational age** |  |  |  |  |  |
| Exposure anytime during pregnancy | 0.354 | 0.640 | 0.798 | 0.264 | 0.549 |
| Exposure in a single trimester | 0.430 | 0.293 | 0.061 | 0.130 | 0.443 |
| Exposure in multiple trimesters | 0.333 | 0.0002 | 0.001 | 0.231 | 0.576 |

Line 63 – heading please and all other headings should be to the left not central, per house style. Please refer to formatting guidelines.

*Response: We have left justified and numbered all headings.*

Refs in the main text need to be in square brackets.

*Response: We have made this change.*

Line 129 – define washout period

*Response: We have now explained that the washout period refers to the period in which there would be ambiguity regarding whether a filled prescription would lead to use during pregnancy (line 224-226).*

Line 252 – dropped. Do you mean excluded?

*Response: We have changed “dropped” to “excluded” (line 456).*

Table 2- it is more conventional for the demographic information to be Table 1. Please reorder and amend any call outs in the text to any re-ordered table.

*Response: We have made this change (line 538).*

Line 291 onward, please remove subscript and use normal text (brackets if needed)

*Response: We have made these changes (line 598-606).*

Line 385 - missingness – perhaps recast – absence?

*Response: Thank you for this suggestion. We have changed “*missingness*” to “*absence of data*” throughout the manuscript and supplement.*

Unless I missed it, please do provide the prespecified analysis plan and a STROBE reporting checklist. Both as supp files. 

*Response: We have provided a STROBE reporting checklist, as well as information on analytic strategy (S1 Appendix).*

**Comments from the reviewers:**

Reviewer #1: This is a well-conducted study on the association between maternal prescribed opioid analgesic use and adverse birth outcomes using population data. The study design, datasets, statistical methods and analyses are mostly adequate and of a good standard, however the presentation and the interpretation of the results could be improved.

*Response: Thank you for your positive feedback. As we have described in detail in response to the specific comments below, we have tried to improve the clarity of our presentation and interpretation of the findings.*

1) The abstract is too long especially in the methods and findings part. Need to be concise to summarise the main findings not all findings and also be able to deliver the key messages. At the moment, it is all over the place with too much details therefore a bit difficult to read.

*Response: We simplified and shortened the abstract by removing some details about single versus multiple-trimester exposure, as well as associations between any POA use during pregnancy and SGA.*

2) Table 2 is a massive table, however it was summarised only by a small paragraph with 5 lines (line 273-277)? and about missing values and their impact? Basically Table 2 is very descriptive, a bit messy to read and not very informative. It would be better to at least have odd ratios in the table to be more informative.

*Response:* We moved the columns describing characteristics of cases exposed in single- and multiple-trimester to the supplement (S13 Appendix). We have also added odds ratios for the associations between the characteristics and any POA exposure during pregnancy (line 538).

3) Missing data. The study is based on complete data analyses therefore sensitivity analysis of missing data is very important. However, Table 2 is not fit for this purpose. A comparison table between complete data and missing data on all the different characteristics and birth outcomes should be included in the main paper or supplementary information. The differences should be seriously discussed and commented as a way of sensitivity analysis.

*Response:* *We have added supplemental analyses assessing the potential bias due missing data (S12 Appendix).*

4) Table 3 is a key and useful table. However, it needs extensive footnotes to explain clearly what exactly these 5 models were adjusted for. It is not practical and reader-friendly for readers to go back to check these in the methods section.

*Response: We have included a footnote explaining all models (line 700-706).*

5) There are quite a few subgroup and sensitivity analyses apart from the main analyses and sometimes it's a bit difficult to follow. Can authors provide clear explanations and logics to why these many sub-analyses are necessary and what they are really for? The same applies to the 5 models.

*Response: When we introduce the variables describing the subgroups, we now briefly describe the purpose of these variables and how they are used in the models (line 224-231). We have now also explained the sensitivity analyses in more depth (line 340-351; 714-769).*

Reviewer #2: This manuscript is a population-based analysis that aims to determine the causal impact of maternal prescribed opioid analgesic use during pregnancy and adverse birth outcomes. I thought this was an excellent analysis and well-written manuscript, and I was particularly impressed with the series of sensitivity analyses addressing various possible biases and looking for ways to fully account for measured and unmeasured confounding.

*Response: Thank you for your positive response to our study.*

Comments:
1.      Abstract conclusion, line 60: Would it be fair to say that the confounding factors are those that are usually unmeasured in other studies, e.g. those that were only accounted for when comparing with unexposed siblings or cousins? I think this could be made clearer in the conclusion.

*Response: Thank you -- this is an important point. In the conclusion section, we have now noted that associates were largely due to unmeasured confounding (line 82).*

2.      Discussion, first paragraph - I think the main findings could be more clearly expressed here. It is a very complex series of analyses, so I understand that they are difficult to condense, but I don't think the main findings, as expressed in the Abstract are clear enough here.

*Response: Thank you for this feedback. We have added more interpretation of the findings to help improve the clarity (line 793-808).*

3.      Could the authors please further justify their reasons for not dealing with the large proportion of missing data through a principled missing data technique such as multiple imputation or inverse probability weighting? The authors state that missingness was weakly associated with exposure status, but what about the association with the outcome? Were there auxiliary variables available (those not already in the adjusted models) that could have been used in multiple imputation?

*Response:* *We have added supplemental analyses assessing the potential bias due missing data (S12 Appendix).*

Minor comments:
- I was sometimes confused by the term 'offspring' and would suggest changing this to 'infants' throughout. When reading about 'exposed and unexposed offspring' I thought initially that this was a sibling comparison, as I was interpreting this as the exposed and unexposed infants being the offspring of the same mother.

*Response: Rather than using the term “offspring,” we now use the terms “births” and “infants” throughout the manuscript.*

Line 89 - Acetaminophen is called paracetamol in Australia (and possibly other countries?). I would suggest putting paracetamol in brackets after the first mention, and perhaps give a very small description as well.

*Response: We now note that paracetamol is another name for acetaminophen (line 163).*

Line 94 - Were the authors able to remove women who used opioids illicitly during the pregnancy or just those on a treatment register?

*Response: While we do not have information on illicit opioid use, we did statistically control for specialist-diagnosed opioid use disorder (line 253).*

Line 127-128 - Should this be 360 to 91 days before conception (not 89)? Otherwise there is a one-day cross-over with the wash-out period.

*Response: Thank you for bringing this to our attention. You are correct. We made this change (line 218).*

Line 168 - Suggest "birthweight 2 standard deviations below the expected fetal weight for gestational age or lower", otherwise sounds like it is less than 2 standard deviations, e.g. 1 SD.

*Response: Thank you for this suggestion to clarify our outcome definition. We have made this change (line 38; line 275).*

Line 193 - Doesn't the null difference between POA-exposed and acetaminophen-exposed offspring only provide support for confounding by indication if it is assumed that acetaminophen is safe and has no influence on the adverse outcomes? I think this needs to be discussed, perhaps in the Methods, with references to support this assumption.

*Response: In the methods section we have more clearly stated that this model assesses the relative safety of the two medications (line 309). When we discuss limitations in the discussion section, we have also now pointed out that a limitation of the comparative safety design is that a null difference could be due to both medications having adverse effects rather than confounding by common indications for the medications (line 848-851).*

Line 214 - 'exposures' should be 'exposure'.

*Response: Thank you for pointing this typo out to us. We have made this correction (line 345).*

Line 216 - what was the reason/justification for the restricted exposure window excluding 3 days before birth?

*Response: We now explain that we restricted the exposure window in case women were prescribed POAs at the end of pregnancy to use after deliver (line 348).*

Line 235-236 - "Fifth, in order to assess if results with exposure in a single trimester and exposure in multiple trimesters would replicate with another exposure also evaluating quantity of exposure,…" I found this phrase difficult to follow. Perhaps "Fifth, in order to assess whether the single versus multiple trimester dose-response results would be replicated with another indicator based on quantity of exposure,…"

*Response: Due to the length of the supplement, we have removed this sensitivity analysis.*

Line 259 - Does the inclusion of 9201 unique mothers mean that siblings with the same father but different mother were included? Can the authors elaborate on why these siblings were included? While siblings with the same father may account for some factors, they do not account for the mother-specific confounders, including ongoing health issues that might affect placental health and risk of preterm, SGA etc. 

*Response: The sibling comparison model compared pregnancies occurring to the same mother. In other words, siblings with the same father but different mother were not analyzed in the same strata. We have clarified that sentence but specifying that the sample of siblings “shared mothers” (line 462).*

Line 265 - Does the prescribed opioid analgesic exposure group exclude those prescribed acetaminophen?

*Response: We did not exclude cases with prescribed acetaminophen from the POA exposure group.*

Line 332 - Include short description of continuous outcome findings.

*Response: Thank you for this suggestion. We have added a short description of these findings (line 757-760).*

Line 349-351 - Aren't the commensurate findings the case for all analyses except for the sibling and cousin comparisons, which showed null findings?

*Response: We clarified that we found converging evidence to suggest that the associations are at least partially due to confounding (line 806-808).*

Reviewer #3: This is an interesting study exploring the relationships between prescription opioid analgesia prescriptions and two birth outcomes, mainly preterm birth (defined as prior to 37 weeks) and small for gestational age (as a proxy for IUGR). The authors used a large robust database and attempted by using multiple analysis to control for as many confounders as they could (including the main issue of confounding by indication, by comparing with acetaminophen prescription and sibling comparisons). This is a vast improvement on other studies which didn't attempt to control for these factors. The authors do a good job of explaining the rationale for the study in the introduction and reviewing the literature to date. The abstract reflects the main findings, the writing is clear, though a bit wordy. The discussion clearly delineates the strengths, weaknesses, and areas for further study. It would be good to use such a large database to look at birth defects.
I would suggest a few changes.

*Response: Thank you so much for your positive feedback about our manuscript.*

Table 2 is very long and some of the variables are quite granular. It is unclear if their initial logistic regression analysis included all of these covariates and found them all to be non-significant (it would be interesting to look at poverty and smoking separately) and then settled on pre-pregnancy exposure and sibling exposure as being the ones that settled out from the logistic regression (and thus redid the analysis using just those variables). I'm not a statistician so I cannot speak to the robustness of the statistical analysis.

*Response: S5 appendix includes all point estimates from adjusted population-wide associations. Not all covariates were non-significant in these models. We now explicitly note in the text that all models that target unmeasured confounding, including the pre-pregnancy model and sibling comparison model, include all covariates that varied between comparison groups (line 328-329).*

I was interested to see that 18-29% of the fathers had prior criminal involvement (if I'm reading that correctly). I think that would influence the outcomes, especially if interpersonal violence is involved.

*Response: Father’s prior criminal involvement is associated with POA exposure (OR=1.37, 95% CI: 1.33, 1.41), as well as both PTB (OR=1.14, 95% CI: 1.11, 1.17) and SGA (OR=1.10, 95% CI: 1.06, 1.14). Therefore, it could serve as a confounder. As such, we included it as a covariate.*

Reference 46 looks like it was glitched in the referencing software and needs to be redone.

*Response: Thank you for pointing this out. We fixed this reference (line 1079).*

There's a typo on page 25 of the supplementary materials.

*Response: Thank you for finding this typo. However, due to the length of the supplement, we have removed this sensitivity analyses.*

Reviewer #4: 
Thank you for the opportunity to review this manuscript.  I found it very informative and extremely well written

My only minor suggestions are: 

Line 63 The estimate of 30% of pregnant women filling opioid prescriptions seems very high, please give more details of this study.

*Response: We have now included more details about this study (line 130-131).*

Line 70/71, can you put in % for use of POA, illicit and medication assisted treatment 

*Response: We have added information on the prevalence of illicit substance use and mediation assisted treatment during pregnancy (line 138-140).*

Main analysis:
There is a great deal of information here. Some subheadings would help here given the many models tested, same comment for sensitivity analysis.

*Response: We have added subheadings.*
